# Capillary–Large Vessel Segmentation on OCTA for Predicting Anti-VEGF Treatment Outcomes in Diabetic Macular Edema

**DOI:** 10.3390/jpm16070341

**Published:** 2026-06-24

**Authors:** Rui-Bin Huang, Jia-Pang Jhang, Bo-Da Huang, Mansour Abtahi, Albert K. Dadzie, Behrouz Ebrahimi, Xincheng Yao, Yi-Ting Hsieh

**Affiliations:** 1 Department of Ophthalmology, National Taiwan University Hospital Hsin-Chu Branch, Hsinchu 300, Taiwan; 2 Department of Ophthalmology, National Taiwan University Hospital, Taipei 100, Taiwan; 3 Department of Ophthalmology, Fu-Jen Catholic University Hospital, New Taipei 243, Taiwan; 4 Department of Biomedical Engineering, University of Illinois Chicago, Chicago, IL 60607, USA; 5 Department of Ophthalmology, School of Medicine, National Taiwan University, Taipei 100, Taiwan

**Keywords:** optical coherence tomography angiography, diabetic macular edema, vessel density, vascular endothelial growth factor

## Abstract

**Objective:** This study aimed to evaluate the predictability of baseline optical coherence tomography angiography (OCTA) metrics utilizing a specialized capillary–large vessel segmentation analysis framework in patients with diabetic macular edema (DME) undergoing anti-vascular endothelial growth factor (anti-VEGF) therapy. **Methods:** Forty-two treatment-naïve eyes with DME receiving three monthly loading anti-VEGF injections were included. Superficial capillary plexus (SCP) images from 3 × 3 mm OCTA scans were processed to isolate the capillary network from the large vessels via image processing. Vessel density and skeleton density were extracted for the total, large-vessel, and capillary components. Multiple linear and logistic regression models were used to identify independent predictors of post-treatment best-corrected visual acuity (BCVA) and “good visual outcome” (≥3-line improvement or final BCVA of 20/40 or better). **Results:** Following three monthly anti-VEGF injections, the mean BCVA significantly improved from 0.57 ± 0.36 to 0.37 ± 0.30 LogMAR (*p* < 0.0001), and the mean central retinal thickness decreased from 424.3 ± 117.7 μm to 316.9 ± 84.7 μm (*p* < 0.0001). The proportion of patients who achieved a good visual outcome was 73.8%. Baseline central retinal thickness was associated with baseline BCVA (*p* = 0.049) but not predictive of post-treatment BCVA (*p* = 0.38) or good visual outcomes (*p* = 0.79). Baseline capillary vessel density was identified as a significant independent predictor of post-treatment BCVA (*p* = 0.024), whereas total and large-vessel metrics were not. Capillary vessel density was also the only significant predictor of good visual outcomes (*p* = 0.044). **Conclusions:** Baseline capillary vessel density is a robust predictor of visual prognosis after anti-VEGF therapy in patients with DME, underscoring the importance of capillary network integrity in functional recovery.

## 1. Introduction

Diabetic retinopathy (DR) is the most common complication of diabetes mellitus [1], and diabetic macular edema (DME) is a leading cause of visual loss in patients with DR [2]. Anti-vascular endothelial growth factor (anti-VEGF) therapies such as ranibizumab and aflibercept have been widely adopted for the treatment of DME, showing efficacy in reducing macular edema and improving visual acuity [3]. However, clinical observations indicate that visual acuity may not improve significantly in all patients, even after the resolution of macular edema. Therefore, reliable biomarkers for predicting treatment outcomes are of paramount importance to determine patient prognosis in clinical practice.

Since its inception in 1991, optical coherence tomography (OCT) has revolutionized ophthalmology by providing non-invasive high-resolution cross-sectional imaging of the retina [1]. As an extension of OCT, OCT angiography (OCTA) has gained prominence as a crucial non-invasive tool, offering detailed insights into the retinal microvascular network with exceptional clarity [4]. In contrast to fluorescein angiography (FA), OCTA noninvasively provides superior resolution of retinal vascular structures, enabling the quantification of microvascular parameters.

The prognostic potential of OCTA biomarkers in predicting treatment response in DME is an area of active research. Some OCTA parameters, such as vessel density and skeleton density, have emerged as potential indicators for forecasting visual recovery following anti-VEGF injections [5,6,7,8]. Our previous study found that baseline parafoveal vessel density within the superficial capillary plexus (SCP) independently predicted visual gain after the initial loading course of ranibizumab [8]; however, other studies present conflicting evidence regarding the predictive value of retinal OCTA metrics [5,6,7]. These discrepancies may be attributed to differences in OCTA image analysis methods. Historically, automated retinal vascular extraction relied heavily on structural intensity-based thresholding or hand-crafted filter banks, which often struggled with the low contrast and high noise characteristic of pathological scans [9,10,11]. Moreover, large vessels and capillaries may have different impacts on visual outcomes; thus, conventional vessel analysis without capillary–large vessel separation may reduce the sensitivity of OCTA in detecting subtle microvascular changes.

To address the limitations of conventional OCTA metrics, a capillary–large vessel segmentation analysis framework was adopted from our previous study on DR [12]. This methodology enables segmentation of the capillary network from the large retinal vessels in the SCP, thus improving the performance of DR classification by extracting features that are specific to large vessels or capillaries in OCTA. This study aimed to evaluate the predictive value of quantitative OCTA perfusion biomarkers, specifically by separating the large vessels and capillaries in the SCP, to determine their effect on visual improvement following anti-VEGF treatment in patients with DME.

## 2. Materials and Methods

### 2.1. Study Population

This study adhered to the tenets of the Declaration of Helsinki and was approved by the Institutional Review Board of National Taiwan University Hospital (No. 202207038RINB). A data-sharing agreement was established between the National Taiwan University Hospital and the University of Illinois, Chicago, for the analysis of de-identified image data. In this retrospective study, we enrolled patients with DME who were treated with intravitreal ranibizumab or aflibercept at National Taiwan University Hospital and received their first injections between October 2015 and December 2021. Only patients ≥ 18 years with a clinical diagnosis of DME confirmed by OCT and who had not received anti-VEGF treatment within the preceding 3 months were included. The diagnostic criteria for DME were DR with focal or diffuse leakage in the macular area documented by FA, and macular edema with the presence of retinal thickening, intraretinal cysts, intraretinal hyperreflective foci, or subretinal fluid as documented by OCT. All patients received three consecutive monthly intravitreal injections of ranibizumab or aflibercept as loading treatment. Patients were excluded if they had other retinal diseases, a history of ocular surgery within the past 6 months, significant media opacities affecting image quality, or severe artifacts that were unadjustable in their OCTA images. OCTA images were obtained at baseline, and best-corrected visual acuity (BCVA) measurements were collected at baseline and 1 month after the third intravitreal injection.

### 2.2. Data Acquisition and Capillary–Large Vessel Differentiation for OCTA

The RTVue XR Avanti System OCT (Optovue, Fremont, CA, USA) was used to obtain 3 × 3 mm macular scans from each subject. The built-in AngioVue software (version 2018.1.1.63) automatically segmented the layers of the SVP, deep capillary plexus (DCP), outer retina, and choriocapillaris, and en face images of the SVP were exported for analysis. Manual corrections were performed for the segmentation errors. The SVP images were then imported into custom-developed MATLAB software Version R2024b (MathWorks, Natick, MA, USA) for capillary–large vessel differential analysis to extract the images of capillaries and large vessels separately, as described previously [12].

**Figure 1:**
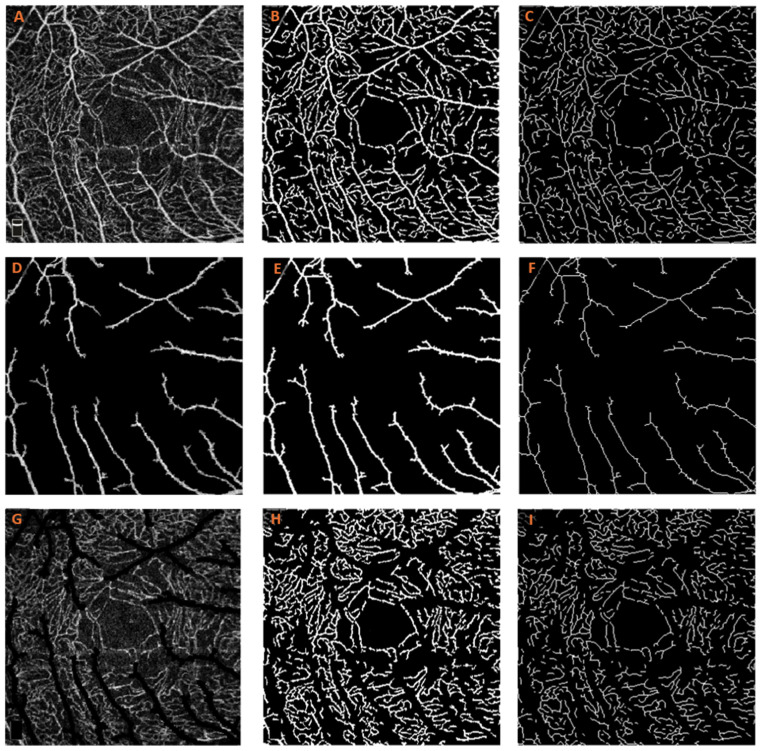
Image processing for capillary–large vessel segmentation for macular optical coherence tomography angiography (OCTA): (**A**) Original OCTA image with total vasculature. (**B**) Binarized image of total vasculature. (**C**) Skeletonized image of total vasculature. (**D**) Image of large vessels only. (**E**) Binarized image of large vessels. (**F**) Skeletonized image of large vessels. (**G**) Images of capillaries. (**H**) Binarized image of capillaries. (**I**) Skeletonized image of capillaries.

To ensure reproducibility, our custom pipeline executed a highly standardized, sequential image-processing chain. Figure 1 shows the image-processing procedure. First, global thresholding was applied to generate a binary vascular map. To extract the large-vessel mask, morphological opening and closing operations with a disk-shaped structuring element (radius of 3 pixels) were employed to effectively remove small capillaries while preserving larger vessels (e.g., arterioles and venules). A capillary-specific image was obtained by subtracting the large-vessel mask from the total vascular map. Following the separation of capillaries and large vessels, the vessel density and skeleton density were calculated for all vessels, large vessels, and capillaries. Vessel density was defined as the percentage of the area occupied by blood vessels, whereas skeleton density was calculated after skeletonizing the vascular map to a single pixel width to represent the linear length of the vasculature, as described previously [8].

### 2.3. Statistical Analysis

Statistical analyses were conducted using SAS software (version 9.4; SAS Institute, Inc., Cary, NC, USA). BCVA was converted to the logarithm of the minimum angle of resolution (logMAR) for analysis. Paired Student’s *t*-test was used to compare BCVA and central retinal thickness (CRT) before and after anti-VEGF injections. To determine the predictive value of baseline OCTA biomarkers, Pearson’s correlation analysis was used to assess the relationship between baseline OCTA biomarkers and baseline BCVA. Multiple regression analysis was performed to identify independent predictors of post-treatment BCVA after adjusting for age and baseline BCVA. To evaluate the clinical significance, patients were further categorized into the “good visual outcome” group (defined as a BCVA improvement of ≥3 lines or a final BCVA of 20/40 or better) or not. Logistic regression was used to determine the association between baseline OCTA biomarkers and the probability of achieving a good visual outcome. Receiver operating characteristic (ROC) curves were drawn for the OCTA biomarkers with significant association, and the areas under the curve (AUCs) were calculated. Statistical significance was set at *p*-value < 0.05. significant.

## 3. Results

### 3.1. Demographic Data and Treatment Outcomes

Forty-two eyes of 32 patients with DME were included in this study. The mean age of the study eyes was 60.1 ± 10.6 (33 to 79) years, and 19 were males and 23 were females. Of the 42 eyes, 15 had moderate non-proliferative diabetic retinopathy (NPDR), 10 had severe NPDR, and 17 had proliferative diabetic retinopathy (PDR). At baseline, the mean logMAR of BCVA was 0.57 ± 0.36, and the mean CRT was 424.33 ± 117.72 μm. A thicker CRT (*p* = 0.049) and older age (*p* = 0.046) were associated with poorer baseline BCVA.

After three monthly loading injections of anti-VEGF, the logMAR of BCVA improved significantly to 0.37 ± 0.30 (*p* < 0.0001), and the CRT significantly decreased to 316.90 ± 84.68 μm after treatment (*p* < 0.0001). A poorer baseline BCVA (*p* < 0.0001) and older age (*p* = 0.020) were associated with poorer BCVA after treatment. Baseline CRT (*p* = 0.38) and post-treatment CRT (*p* = 0.062) did not significantly correlate with post-treatment BCVA. Thirty-one eyes (73.8%) achieved “good visual outcome,” while the baseline CRT was not correlated with it (*p* = 0.79).

Regarding therapeutic interventions, 35 eyes (83.3%) were treated with intravitreal ranibizumab, and 7 eyes (16.7%) received intravitreal aflibercept based on real-world clinical allocation and physician discretion. There were no significant differences between the ranibizumab and aflibercept groups in terms of age, baseline logMAR BCVA, baseline CRT, post-treatment BCVA, or post-treatment CRT (*p* > 0.05 for all) (Supplementary Table S1).

### 3.2. Correlations Between Baseline OCTA Biomarkers and BCVA

Multiple linear regression analysis was performed to evaluate the correlations between baseline OCT biomarkers and BCVA with age and baseline CRT adjusted in the models. None of the baseline OCTA vascular density or skeleton density showed a significant correlation with baseline BCVA (*p* > 0.05) (Table 1).

### 3.3. Predicting Post-Treatment BCVA with Baseline OCTA Biomarkers

Multiple linear regression analysis was performed to predict post-treatment BCVA with baseline OCTA biomarkers, adjusted for age and baseline BCVA in the models. After adjusting for age and baseline BCVA, baseline capillary vessel density was found to be a significant independent predictor of post-treatment BCVA (*p* = 0.044); the higher the baseline capillary density, the better the post-treatment BCVA. Neither the vessel density of the total vessels nor that of the large vessels correlated with post-treatment BCVA. Skeleton density parameters did not correlate with post-treatment BCVA (*p* > 0.05) (Table 2).

### 3.4. Predicting Good Visual Outcome with Baseline OCTA Biomarkers

**Figure 2:**
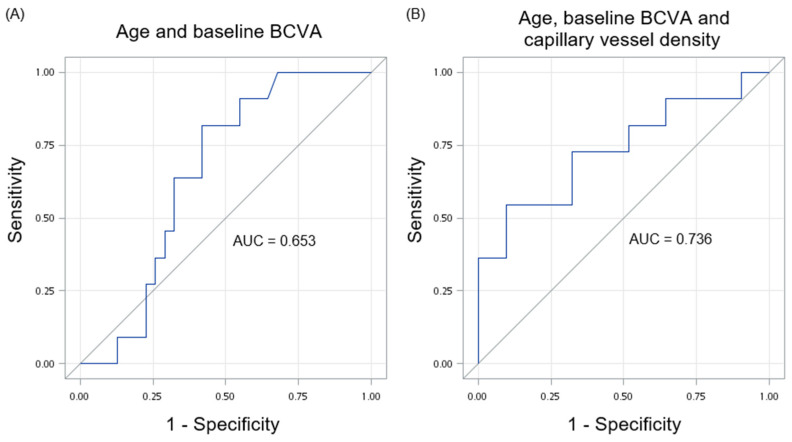
The receiver operating characteristic (ROC) curves for predicting good visual outcome with (**A**) age and baseline BCVA; (**B**) age, baseline BCVA, and capillary vessel density.

**Figure 3:**
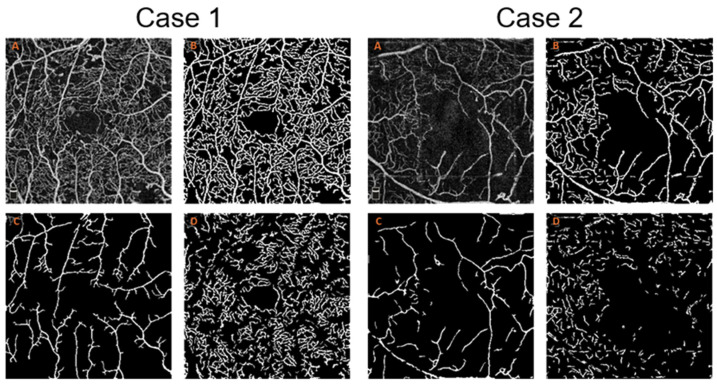
Two representative cases. Case 1 was a 49-year-old man who was diagnosed with proliferative diabetic retinopathy and diabetic macular edema in both eyes. In the right eye, the baseline central retinal thickness (CRT) was 350 μm, and the best-corrected visual acuity (BCVA) was 20/60. After three monthly loading injections of intravitreal ranibizumab, the CRT decreased to 274 μm, and BCVA improved to 20/25. The baseline capillary vessel density was 58.73. Case 2 was A 55-year-old male with the diagnosis of proliferative diabetic retinopathy and diabetic macular edema in both eyes. For the left eye, the baseline CRT was 314 um, and the BCVA was 20/200. After three loading injections of intravitreal ranibizumab, the CRT decreased to 219 um, but the vision only improved to 20/133. The capillary vessel density at baseline was 33.44. In both cases: (**A**) original OCTA image with total vasculature; (**B**) binarized image of total vasculature; (**C**) binarized image of large vessels; (**D**) binarized image of capillaries.

To predict “good visual outcome,” multiple logistic regression analysis with adjustment for age and baseline BCVA was employed. In this predictive model, capillary vessel density was identified as the sole significant predictor of good treatment outcome (*p* = 0.044); the higher the baseline vessel density, the higher the chance of achieving a good treatment outcome. Figure 2 shows the ROC curves for predicting a good visual outcome with age, baseline BCVA, and capillary vessel density, and the AUC increased to 0.736 from 0.653 when using only age and baseline BCVA. Neither the vessel density of total vessels or large vessels nor their skeleton density was significantly correlated (*p* > 0.05) (Table 3). Figure 3 shows two representative cases. Both cases had similar baseline CRT and BCVA, and macular edema subsided after three monthly loading injections of ranibizumab in both eyes. However, the post-treatment visual outcomes differed substantially, highlighting the potential impact of baseline capillary vessel density on visual recovery.

## 4. Discussion

This study evaluated the predictive value of quantitative OCTA biomarkers, specifically focusing on the segregation of capillaries and large vessels, for visual outcomes following anti-VEGF treatment in patients with DME. The results of this study demonstrated that while baseline CRT correlated with initial visual acuity, it failed to predict final functional outcomes. Instead, through the differentiation of capillaries and large vessels, the vessel density of capillaries in the SVP was identified as a significant independent predictor of post-treatment visual acuity and the likelihood of achieving a “good visual outcome.”

The predictability of baseline OCTA parameters for visual outcomes after anti-VEGF treatment for DME has been studied previously. Our previous study found that baseline parafoveal vessel density within the SCP independently predicted visual gain after the initial loading course of ranibizumab [8]. However, Lee et al. [6] reported that a significant compromise of the deep capillary plexus (DCP), and not the SCP, was an indicator of poor response. In this study, we chose SCP but not DCP for analysis. Although DCP may be more relevant in DME and macular ischemia, quantitative DCP vessel density measurement in DME is technically challenging because macular edema can cause segmentation errors [13], signal attenuation from cystoid spaces [11], and projection artifacts from the superficial plexus [14], all of which may disproportionately affect deeper-layer OCTA metrics. Some prior studies have shown that vessel density measurements in DCP have poorer repeatability in eyes with retinal vasculopathy and/or cystoid macular edema [15,16]. Therefore, in the present study, we focused on the SCP, where capillary–large vessel separation is technically feasible and less confounded by edema-related deep-layer artifacts. In addition to the impact of different anti-VEGF agents in different studies, it is possible that large vessels appearing in the OCTA images of the SCP would interfere with the measurement of capillary vessel density, which represents the extent of macular ischemia. This study segmented the capillaries and large vessels in SCP OCTA images to measure the effects of capillaries and large vessels separately. It was discovered that only the capillaries, but not the large vessels or total vessels, had a predictive effect on visual prognosis after anti-VEGF treatment. This suggests that the actual area of perfused capillaries at baseline is the most critical determinant of a macula’s potential for functional recovery once the edema is resolved. This aligns with the concept that capillary bed health is a direct proxy for the preservation of the neurosensory retina. The lack of correlation in the total vascular network is likely due to the confounding presence of large vessels, which remain relatively stable in diameter and do not reflect the subtle microcirculatory ischemia characteristics of DR. This study shows the superiority of capillary-specific metrics over total vascular metrics in predicting visual prognosis.

Consistent with the previous literature, we observed a significant reduction in CRT and improvement in BCVA after three monthly anti-VEGF injections. However, this study showed that baseline CRT was not predictive of the final BCVA (*p* = 0.6188). This “dissociation” between anatomical severity and functional impairment is a well-documented challenge in the management of DME. Although anti-VEGF therapy effectively reduces vascular permeability and resolves intraretinal fluid, the ultimate visual recovery depends on the underlying integrity of the microvasculature and photoreceptors. This study bridges this gap by demonstrating that capillary density can serve as a robust biomarker for identifying patients who possess sufficient “vascular reserves” to benefit functionally from anatomical drying.

Interestingly, while baseline capillary vessel density was predictive of visual outcomes after treatment, neither capillary nor total vessel density was significantly correlated with baseline BCVA. Our previous study found no correlation between vessel density and visual acuity in eyes with DME [17]. However, other studies have reported that eyes with DME exhibit significantly more impaired vascular integrity, including lower capillary vessel density and a larger foveal avascular zone, than diabetic eyes without DME [18,19]. This may be explained by the overwhelming impact of macular edema (CRT on initial vision. As shown in our determinants of baseline BCVA, CRT, and age were the primary drivers of poor vision. It appears that the physical distortion caused by fluid accumulation masks the underlying relationship between capillary density and visual function. Only after the fluid is removed does the importance of capillary perfusion density become evident in the final visual outcome.

Another notable observation in this study was the performance of the skeleton density. Although skeleton density is often touted as a more sensitive measure of early capillary loss because it reduces vessels to a single-pixel width to minimize the impact of vessel dilation, it did not reach statistical significance in this study’s predictive models (*p* = 0.19). Unlike area-based vessel density, skeleton density reduces the vascular network to a single-pixel width, thereby minimizing the confounding influence of vessel caliber and providing a more sensitive measure for early capillary dropout [20,21]. Our previous study demonstrated that lower skeleton density rather than vessel density in the deep capillary plexus significantly correlates with poorer baseline visual acuity in patients with DME, highlighting its role as a key indicator of macular ischemia [17]. However, this finding was observed only in the deep capillary plexus, not in the superficial capillary plexus. Although in another study targeting the prediction of visual outcomes after anti-VEGF therapy, skeleton density did not emerge as a superior predictor. As discussed by Pramil et al. [20], the presence of macular edema often distorts the retinal architecture and degrades the image contrast, which can lead to significant automated segmentation errors and “shadowing artifacts” from the intraretinal fluid. Lazăr et al. [21] also noted that because skeletonization relies on precise pixel-level extraction, it is particularly vulnerable to this “mechanical noise” compared to area-based vessel density. The findings of this study suggest that while skeleton density is theoretically a more sensitive marker for chronic ischemic changes, capillary-specific vessel density may offer a more robust and noise-resistant biomarker for predicting functional recovery in the acute treatment-naïve phase of DME. In DME, where the vessel caliber may be altered by both the disease process and the presence of edema, the actual “area” of perfusion, represented by vessel density, may be a more clinically relevant biomarker for predicting the survival of neuronal tissue than the linear length of the vasculature.

This study had several limitations that warrant consideration. First, the sample size of 42 eyes was relatively small, which may have limited the generalizability of the findings and the statistical power to detect subtle differences in other skeletal metrics and carried a potential risk of overfitting. To ensure model stability, we restricted covariates to two established clinical factors (age and baseline BCVA), adhering to the rule-of-thumb for the variable-to-sample size ratio. The model’s robustness is supported by an adjusted R2 of 0.549, an AUC of 0.736 for capillary vessel density, and low variance inflation factors (VIFs), confirming that multicollinearity did not confound our results. While these metrics indicate that baseline capillary vessel density is a stable, independent predictor of visual prognosis, larger cohorts are required for validation. Second, the study population was recruited from a single institution with a concentrated age range, potentially introducing selection bias. Third, there was heterogeneity in the specific anti-VEGF agents, which reflected a real-world setting where patients received either ranibizumab or aflibercept. Preliminary comparisons showed no significant differences in visual or anatomical outcomes between the two drug groups. To avoid over-parameterizing our predictive models and risking statistical overfitting, the specific anti-VEGF agent was excluded from the multiple regression analysis. Future large-scale studies should investigate whether different agents interact differently with perfusion biomarkers. Furthermore, this study focused on the 3-month loading phase, restricting findings to short-term outcomes. As DME requires long-term maintenance protocols (e.g., treat-and-extend or pro re nata), whether baseline capillary density predicts long-term visual sustainability remains to be determined. Future longitudinal investigations are required to validate the long-term prognostic value of our segmentation framework. Finally, due to the retrospective design, data regarding the exact duration of diabetes and glycemic control (HbA1c) were unavailable, which may influence long-term retinal chronicity and response capacity.

## 5. Conclusions

In conclusion, this study highlights the clinical utility of segmenting the capillary network from large vessels in OCTA analysis of DME. Capillary vessel density at baseline is a robust independent predictor of visual recovery after anti-VEGF therapy for DME, outperforming CRT and traditional total vascular metrics. Integrating capillary–large vessel segmentation analysis into clinical practice could significantly enhance personalized management of DME by providing clinicians with a tool to better forecast treatment responses and manage patient expectations.

## Figures and Tables

**Table 1:** Multiple linear regression analysis for baseline best-corrected visual acuity with adjustment for age and baseline central retinal thickness.

	Coefficient	*p* Value	Adjusted R^2^	VIF
Vessel density				
Total	−0.0068	0.26	0.167	1.024
Large vessels	−0.0380	0.14	0.188	1.044
Capillaries	−0.0043	0.44	0.152	1.062
Skeleton density				
Total	−0.0403	0.29	0.163	1.004
Large vessels	−0.1057	0.21	0.173	1.055
Capillaries	−0.0316	0.38	0.155	1.148

VIF: variance inflation factor.

**Table 2:** Multiple linear regression analysis for post-treatment best-corrected visual acuity with adjustment for age and baseline best-corrected visual acuity.

	Coefficient	*p* Value	Adjusted R^2^	VIF
Vessel density				
Total	−0.0074	0.060	0.530	1.040
Large vessels	−0.0029	0.86	0.484	1.094
Capillaries	−0.0077	0.024	0.549	1.026
Skeleton density				
Total	−0.0277	0.27	0.500	1.035
Large vessels	−0.0054	0.92	0.483	1.076
Capillaries	−0.0397	0.19	0.507	1.171

VIF: variance inflation factor.

**Table 3:** Multiple logistic regression analysis for good visual outcome with adjustment for age and baseline best-corrected visual acuity.

	Coefficient	*p* Value	AUC	VIF
Vessel density				
Total	−0.0819	0.087	0.698	1.040
Large vessels	−0.0032	0.99	0.651	1.094
Capillaries	−0.0852	0.044	0.736	1.027
Skeleton density				
Total	−0.3101	0.29	0.636	1.035
Large vessels	−0.0837	0.89	0.645	1.076
Capillaries	−0.3251	0.23	0.655	1.171

AUC: area under the curve; VIF: variance inflation factor.

## Data Availability

Data will be available upon reasonable request from the corresponding author.

## Supplementary Materials

The following supporting information can be downloaded at: https://www.mdpi.com/article/10.3390/jpm16070341/s1, Table S1: Comparison between eyes receiving ranibizumab and those receiving aflibercept.

## Abbreviations

The following abbreviations are used in this manuscript:

OCTA	Optical coherence tomography angiography
DME	Diabetic macular edema
Anti-VEGF	Anti-vascular endothelial growth factor
SCP	Superficial capillary plexus
DR	Diabetic retinopathy
DCP	Deep capillary plexus
