# Supplementary material for: Capillary–Large Vessel Segmentation on OCTA for Predicting Anti-VEGF Treatment Outcomes in Diabetic Macular Edema
Source: J Pers Med. 2026 Jun 24;16(7):341. doi: 10.3390/jpm16070341 (PMC13413176; doi:10.3390/jpm16070341)
Supplement: Supplementary file 1 [file jpm-16-00341-s001.zip › jpm-4288335-supplementary.pdf]

Supplementary Table S1. Comparison between eyes receiving ranibizumab and those receiving aflibercept.

	Ranibizumab	Aflibercept	*P* value
Case number	35	7	
Age	59.9 ± 9.5	61.0 ± 15.9	0.40
Baseline logMAR of BCVA	0.59 ± 0.35	0.48 ± 0.39	0.31
Baseline CRT	428.3 ± 79.6	404.6 ± 114.7	0.54
Post-treatment logMAR of BCVA	0.38 ± 0.31	0.31 ± 0.23	0.64
Post-treatment CRT	315.8 ± 87.9	322.3 ± 72.0	0.65
